# Antibacterial Effect of Amino Acid–Silver Complex Loaded Montmorillonite Incorporated in Dental Acrylic Resin

**DOI:** 10.3390/ma14061442

**Published:** 2021-03-16

**Authors:** Kumiko Yoshihara, Noriyuki Nagaoka, Aya Umeno, Akinari Sonoda, Hideki Obika, Yasuhiro Yoshida, Bart Van Meerbeek, Yoji Makita

**Affiliations:** 1 Health and Medical Research Institute, National Institute of Advanced Industrial Science and Technology (AIST), Kagawa 761-0395, Japan; siraume2003aka@gmail.com (A.U.); a.sonoda@aist.go.jp (A.S.); h-obika@nifty.com (H.O.); y-makita@aist.go.jp (Y.M.); 2 Department of Pathology & Experimental Medicine, Dentistry and Pharmaceutical Sciences, Graduate School of Medicine, Okayama University Hospital, Okayama University, Okayama 700-8558, Japan; 3 Advanced Research Center for Oral and Craniofacial Sciences, Okayama University Dental School, Okayama 700-8558, Japan; nagaoka@okayama-u.ac.jp; 4 Department of Biomaterials and Bioengineering, Faculty of Dental Medicine, Hokkaido University, Hokkaido 060-8586, Japan; yasuhiro@den.hokudai.ac.jp; 5 KU Leuven (University of Leuven) Department of Oral Health Research, BIOMAT & University Hospitals Leuven, B-3000 Leuven, Belgium; bart.vanmeerbeek@kuleuven.be

**Keywords:** montmorillonite, amino acid, antibacterial, *Streptococcus mutans*, nuclear magnetic resonance, X-ray diffraction

## Abstract

Several dental materials contain silver for antibacterial effect, however the effect is relatively low. The reason for the lower antibacterial efficacy of silver is considered to be the fact that silver ions bind to chloride ions in saliva. To develop new effective silver antibacterial agents that can be useful in the mouth, we synthesized two novel amino acid (methionine or histidine)–silver complexes (Met or His–Ag) loaded with montmorillonite (Mont) and analyzed their antibacterial efficacy. At first the complexes were characterized using nuclear magnetic resonance (NMR), and amino acid–Ag complex-loaded Mont (amino acid–Ag–Mont) were characterized using X-ray diffraction (XRD) and scanning electron microscopy (SEM). The antibacterial efficacy of these materials in dental acrylic resin was then investigated by bacterial growth measurement using a spectrophotometer. As controls, commercially available silver-loaded zeolite and silver-zirconium phosphate were also tested. Dental acrylic resin incorporating His–Ag–Mont strongly inhibited *Streptococcus mutans* growth. This was explained by the fact that His-Ag complex revealed the highest amounts of silver ions in the presence of chloride. The structure of the amino acid–Ag complexes affected the silver ion presence in chloride and the antibacterial efficacy. His–Ag–Mont might be used as antibacterial agents for dental materials.

## 1. Introduction

Silver ions (Ag^+^) have long been known to be effective against a broad range of microorganisms [1]. The broad-spectrum antimicrobial properties of silver encourage its use in biomedical applications, water and air purification, food production, cosmetics, clothing, and numerous household products [2].

In dentistry, major diseases such as caries and periodontitis occur due to bacterial infection. Silver compounds have been used to prevent these diseases from as early as the 1840s, when silver nitrate was used to reduce the incidence of caries in the primary dentition. Silver nitrate, silver fluoride, and silver diamine fluoride have been used for dental application or in addition to dental restorative materials [3,4,5,6]. Nevertheless, the antibacterial effects of these compounds may be lower than expected because silver ions easily interact with chloride in a saliva solution [7]. To obtain greater antibacterial effects, silver nanoparticles show more efficient antimicrobial properties due to their large surface area [8,9,10]. However, increasing consumption of silver products leads to worries about human and environmental toxicity and silver resistance in bacteria [5,11]. Therefore, the effectiveness of antibacterial materials with low concentrations of release remains to be investigated.

Other researchers found that silver(I) complexes with donor atoms such as nitrogen or oxygen have a wide effective spectrum of antimicrobial activity [6,7,8,9,10,11,12,13]. For amino acid–silver complex materials, several amino acids such as histidine [14], arginine, glutamic acid [15,16], and aspartic acid [17,18], have been investigated. Each amino acid revealed different antibacterial effects. In our previous study, we investigated the concentration of Ag^+^ in several amino acid–silver complexes in sodium chloride and marine water. The histidine–amino acid complex and methionine-amino acid complex showed higher Ag^+^ ion concentration in high–Cl^−^ marine water [19].

Safety is most important for applying materials for dental application. Essential amino acids exist in food and the human body, and people take them as supplements. Therefore, essential amino acids are not toxic to use for dental applications. However, no previous report has examined whether these silver-amino acid complexes can be used for dental application.

In this study, we investigated the antibacterial effect of amino acids-silver in dental acrylic resin. When dental acrylic resin is hardened after the powder and liquid react, it is difficult to release antibacterial materials from dental acrylic resin. In order to release amino acids–silver, they were loaded onto montmorillonite. Montmorillonite has a layered structure and is often used as a material on which other molecules/compounds [20,21]. For example, the copper nanoparticle loaded montmorillonite can release copper ions and revealed an antibacterial effect not only on contact with materials but also in the surrounding medium [22]. The objective of this study was to investigate differences in the amino acid–silver complex on antibacterial efficacy. The null hypothesis of this study was that the different silver compounds would show no differences in their antibacterial effects.

## 2. Materials and Methods

### 2.1. Synthesis of Amino Acid–Silver Complexes Loaded onto Montmorillonite

We mixed 10 mM essential amino acid (histidine or methionine) solution with 4 mM AgNO_3_ solution at a molar ratio of 10:1 = amino acid: Ag^+^ to form the amino acid–silver complex. Subsequently, 0.5 g of montmorillonite was added to this solution and mixed for 15 h at 50 °C. Met–Ag–Mont (methionine–silver–montmorillonite) and His–Ag–Mont (histidine–silver–montmorillonite) were centrifuged, the supernatant was removed, washed with a distilled water, and then the pellet was dried for 5 h at 50 °C.

### 2.2. Resin Sample Preparation

As antibacterial materials, montmorillonite, Met–Ag–Mont, His–Ag–Mont, silver-zeolite (AJION, Sinanen Zeomic Co. Ltd., Nagoya, Japan), and a silver-based inorganic antimicrobial agent (NOVARON AG300, Toagosei, Tokyo, Japan) were used. These materials were added to dental acrylic resin powder at 0.01, 0.02, 0.03, and 0.05 w% (Unifast lll, GC Tokyo, Japan). The powder was mixed with a regulated amount of Unifast liquid. The acrylic resin was put in a 10 mm diameter, 2 mm thickness silicon mold and kept for 5 min. The surface of the acrylic resin plate was polished using 15 μm diamond rapping film.

### 2.3. Nuclear Magnetic Resonance (NMR) Measurement

In order to measure nuclear magnetic resonance (NMR), each amino acid-silver complex was dissolved in D-water. We recorded the ^1^H and ^13^C NMR spectra at room temperature on an NMR spectrometer (UNITY INOVA400NB, Varian Japan, Tokyo, Japan) at 399.78 and 100.53 MHz, respectively.

### 2.4. Scanning Electron Microscopy (SEM)

In order to disclose the structure of each antibacterial material tested in this study, montmorillonite, Met–Ag–Mont, His–Ag–Mont, silver–zeolite (AJION, Sinanen Zeomic Co. Nagoya, Japan), and silver zirconium phosphate (NOVARON, Toagosei Co. Ltd., Tokyo, Japan) were observed using a field-emission scanning electron microscope (Fe-SEM: Topcon DS-720, Topcon, Tokyo, Japan).

### 2.5. X-Ray Diffraction (XRD)

The crystal phases of the samples were identified using an X-ray powder diffractometer (CuKα; 1.54 Å, RINT 2500, Rigaku, Osaka, Japan) operating under 40 kV acceleration and 200 mA current at a scanning rate of 0.02° per s.

### 2.6. Bacteria

*Streptococcus mutans* (*S. mutans*, ATCC25175), stored at −80 °C, was subcultured on blood agar plates (37 °C, 5% CO2). Colonies from these blood agar plates were cultivated overnight in brain heart infusion broth (BHI, EIKEN CHEMICAL CO., Tokyo, Japan), and the obtained liquid cultures were used for the experiments.

### 2.7. Bacterial Growth Spectrophotometry

To select relevant concentrations of monomers, growth was first assessed by spectrophotometry. Liquid overnight cultures were centrifuged, and bacteria were resuspended in fresh BHI broth. The concentration was adjusted to 1 × 10^6^ colony-forming units (CFU)/mL spectrophotometrically at a wavelength of 600 nm (BioSpectrometer Basic, Eppendorf, Hamburg, Germany). Three disks per material were placed in a 24-well plate (Costar 24 well, Corning, NY, USA), upon which 2 mL of bacterial suspension was added. As positive and negative controls, respectively, three wells did not receive a material disk, and another three wells did not receive a bacterial suspension. The absorbance was measured for 36 h at 600 nm and 37 °C using a microplate reader (POLARstar Omega, BMG LABTECH, Ortenberg, Germany). This procedure was repeated three times (*n* = 3 sites on 3 disks) [23].

### 2.8. Silver Ion Measurement in Different Concentrations of Chloride Ion Solution

Silver ion concentration was measured at different concentrations of chloride ion solution (0, 25, 50, 100, 250, 580, and 1000 ppm) using atomic absorption spectrometry (AAS: AAnalyst 300; Perkin-Elmer, Waltham, MA, USA). To confirm the stability of the Ag^+^, the Ag^+^ concentration change of His–Ag, Met–Ag, or Ag in 6 mM and 612 mM of Cl^−^ was also measured for 25 h.

## 3. Results and Discussion

### 3.1. Montmorillonite-Loaded Amino Acid–Silver Complex Preparation

In order to synthesis of amino acid (histidine or methionine)–silver complex, we used a 10 mM essential amino acid solution was mixed with 4 mM AgNO_3_ solution at a molar ratio of 10:1 = amino acid: Ag^+^ to form the amino acid-silver complex. As preliminary test, we prepared different molar ratio solution of Met/Ag = 0, 1, 3, 6 and 10. Then 4, 135, 546 mmol/L of NaCl was added to the solution. The highest silver ion concentration was recorded for Met/Ag = 10 at all NaCl solution. These results showed higher concentration of methionine revealed higher silver ion. This may can explain that the formation of amino acid-silver complex suppressed deposition of silver chloride. From these data, we used 4 mM AgNO_3_ solution at a molar ratio of amino acid: Ag ^+^ = 10:1 in this study. Furthermore, we loaded the amino acid–silver complex with montmorillonite to release this ability in hardened Poly(methyl methacrylate) (PMMA) dental resin.

### 3.2. NMR Measurement

**Figure 1:**
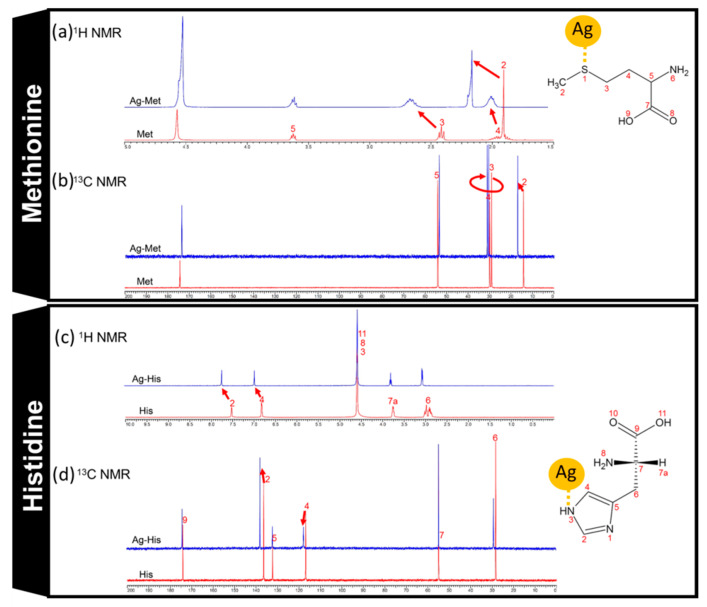
(**a**) 1H nuclear magnetic resonance (NMR) spectra of methionine and methionine–Ag. (**b**) ^13^C NMR spectra of methionine and methionine–Ag. (**c**) 1H NMR spectra of histidine and histidine–Ag. (**d**) ^13^C NMR spectra of histidine and histidine–Ag.

First, we confirmed the structure of the silver-amino acid complexes using NMR. The ^1^H and ^13^C NMR spectra of methionine and histidine are shown in Figure 1a–d. The peaks are assigned to the chemical structures with the corresponding number. The silver-added samples were also measured under the same conditions. For methionine, the number-2 and -3 protons are shown in the larger shift for ^1^H NMR (Figure 1a). Number-2 and 3 carbons are seen as the larger shift for ^13^C NMR (Figure 1b). In the case of histidine, the number-2 and -4 protons are shown in the larger shift for ^1^H NMR (Figure 1c). Number-2 and -4 carbons are observed in the larger shift for ^13^C NMR (Figure 1d). These effects were caused by interactions with the neighboring silver.

In the case of methionine, the Ag–S interaction affected the neighboring ^1^H and 13C peaks (Figure 1a,b). In the case of histidine, the Ag–N (no. 3 or no. 1) interaction affected the neighboring ^1^H and ^13^C peaks (Figure 1c,d). Therefore, silver forms complexes with these amino acids in aqueous solution at this concentration.

### 3.3. Scanning Electron Microscope (SEM) Observation and X-ray Diffraction (XRD) Analysis

**Figure 2:**
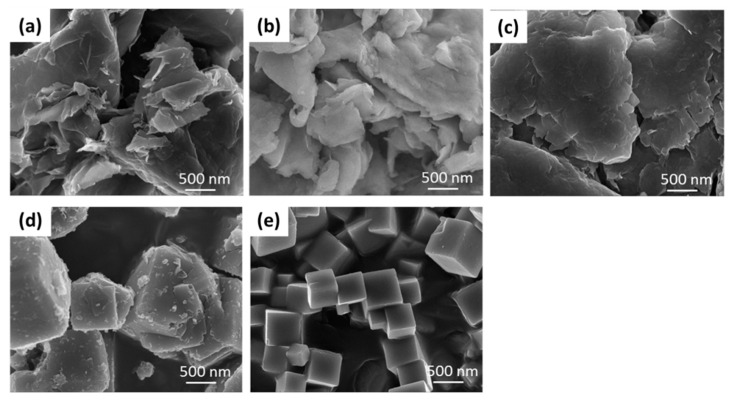
Scanning electron microscopy (SEM) images of montmorillonite (**a**), His–Ag–Mont (**b**), Met–Ag–Mont (**c**), silver zeolite (AJION) (**d**), and silver zirconium phosphate (NOVARON) (**e**).

Figure 2 shows SEM images of montmorillonite (a), His–Ag–Mont (b), Met–Ag–Mont (c), silver zeolite (AJION) (d), and silver zirconium phosphate (NOVARON) (e). Montmorillonite and both amino acid–silver–Mont complexes showed plate-like structures. AJION and NOVARON showed around 500 nm cubic structures.

**Figure 3:**
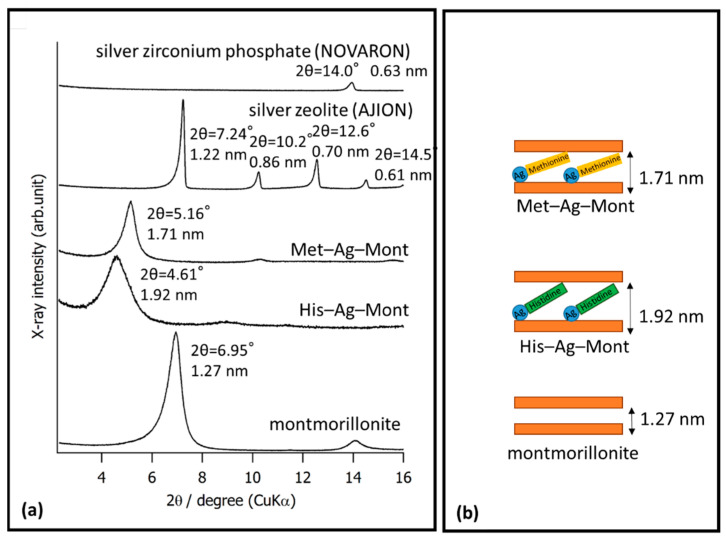
(**a**) XRD analysis of montmorillonite, His–Ag–Mont, Met–Ag–Mont, silver zeolite (AJION), and silver zirconium phosphate (NOVARON). (**b**) Schematic diagram of montmorillonite, His–Ag–Mont, Met–Ag–Mont.

Figure 3a shows the XRD pattern. Montmorillonite revealed a strong peak at 2θ = 6.95° (d = 1.27 nm) and a weak peak at 14.1° (d = 6.29 nm). Met–Ag–Mont revealed a strong peak at 2θ = 5.16° (d = 1.71 nm) and a weak peak at 10.3° (d = 0.86 nm). His–Ag–Mont revealed a strong peak at 2θ = 4.61° (d = 1.92 nm).

When the antibacterial agent cetylpyridinium chloride (CPC) was incorporated with dental resin, the antibacterial effect was revealed only on the surface due to CPC immobilized in resin matrix [24]. Montmorillonite is often used for loading inorganic materials [20,21,22,25]. In order to release Ag^+^ for this study, we used montmorillonite for loading the materials. SEM observation of montmorillonite revealed a layered structure. XRD confirmed that His–Ag–Mont and Met–Ag–Mont were loaded into layers of montmorillonite (Figure 3b) [26]. AJION showed several peaks, including ones at 2θ = 7.24° (d = 1.22 nm), 10.2° (d = 0.86 nm), 12.6° (d = 0.70 nm), and 14.5° (d = 0.61 nm), assigned to type A zeolite (ICDD; International Centre for Diffraction Data). AJION was reported to contain 2.5% (*w*/*w*) of Ag^+^ bound electrostatically to synthetic type-A zeolite [27]. NOVARON revealed a peak at 14.0° (d = 0.63 nm), assigned to sodium zirconium phosphate (ICDD). NOVARON was zirconium phosphate containing Ag^+^ in the crystal structure [28].

### 3.4. Bacterial Growth

In this study, we aimed to develop efficient, longer-lasting antibacterial silver agents for dental application. The chloride concentration of saliva is known to be 20–30 mM [29]. To determine the antibacterial efficacy of the silver agents against *S. mutans*, we used BHI medium for bacterial growth. BHI contains a chloride concentration of 25 mM, nearly the same as saliva.

**Figure 4:**
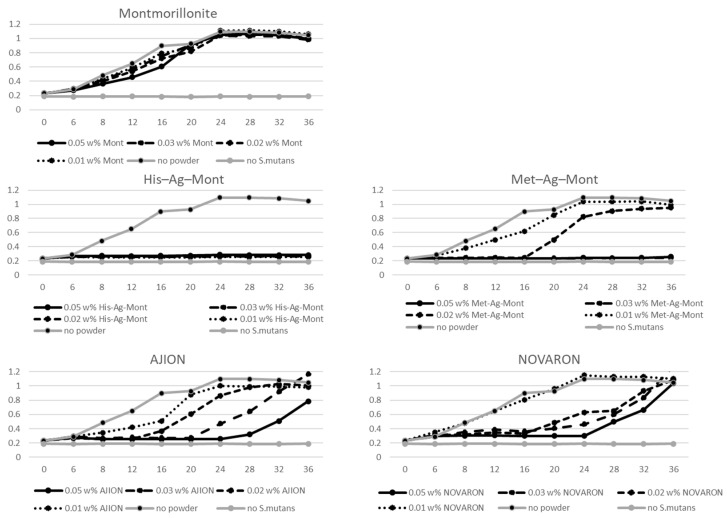
*Streptococcus mutans* growth curves for dental acrylic resin incorporating different amounts of montmorillonite, His–Ag–Mont, Met–Ag–Mont, silver zeolite (AJION), and silver zirconium phosphate (NOVARON).

Figure 4 showed that Streptococcus mutans growth curves for dental acrylic resin incorporating different amounts of montmorillonite. His–Ag–Mont inhibited *S. mutans* growth, whereas acrylic with any concentration of montmorillonite did not inhibit *S. mutans* growth. Acrylic with 0.05 w% AJION showed longer inhibition of *S. mutans* growth, but the optical density (OD) measured by spectrophotometry increased after 28 h. A higher concentration of NOVARON also inhibited some bacterial growth; however, all concentrations of NOVARON revealed bacterial growth after 36 h. Acrylic incorporating 0.03 and 0.05 w% Met–Ag–Mont showed complete inhibition of *S. mutans* growth. In the case of His–Ag–Mont, all studied concentrations in acrylic resin completely inhibited bacterial growth. Therefore, the null hypothesis of this study that the different silver compounds would show no differences in antibacterial effects was rejected.

OD measurement revealed that each silver compound had a different antibacterial effect. NOVARON has an antibacterial effect against *S. mutans*, and the minimum inhibitory concentration (MIC) and the minimum bactericidal concentration (MBC) of NOVARON against *S. mutans* were found to be 40 μg/mL [24]. Kiriyama et al. revealed that dental acrylic resin incorporating 0.5 w% NOVARON inhibited *S. mutans* growth. The results showed a strong correlation between the amount of eluted silver ions and a reduction in the number of colony-forming units (CFUs) [28]. Kawahara et al. revealed that silver-zeolite AJION inhibited the growth of bacteria when tested under anaerobic conditions. However, the MIC value of silver–zeolite in BHI broth was greater than that in water. These authors suggested that proteins and chloride in BHI probably inactivated a significant portion of the Ag^+^ released from silver zeolite [27]. There are two possible mechanisms for the antibacterial effect for silver compounds: first, bacterial cells are inhibited for several functions or are damaged due to silver ions from the silver zeolite. Another mechanism is the generation of reactive oxygen species, due to the inhibition of respiratory enzymes by silver ions, which attack the cell itself [29,30]. Therefore, silver ion concentration is important for achieving an antibacterial effect. Compared to commercially available materials such as AJION and NOVARON, His–Ag–Mont revealed a particularly strong antibacterial effect. This was due to a different silver ion concentration in BHI medium due to the presence of Cl^−^.

All samples revealed many small black spots on the surface after 36 h of bacterial growth measurement. These black spots were due to the formation of silver oxide and silver sulfide [31,32,33,34,35,36].

### 3.5. Silver Ion Measurement in Different Concentrations of Chloride Ion Solution

**Figure 5:**
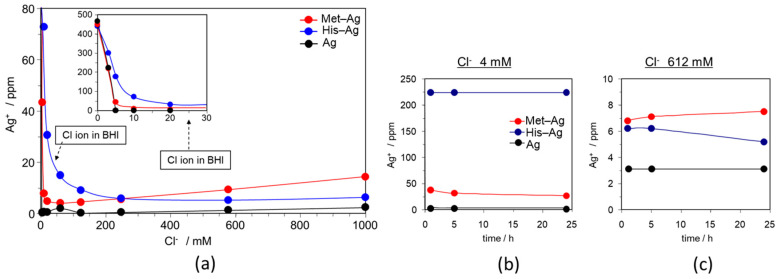
Silver ion measurements using atomic absorption spectrometry (AAS). (**a**) Silver ion concentration of Met–Ag, His–Ag, and Ag in different concentrations of chloride. (**b**) Silver ion concentrations of Met–Ag, His–Ag, and Ag over time in 4 mM Cl^−^ solution. (**c**) Silver ion concentrations of Met–Ag, His–Ag, and Ag over time in 612 mM Cl^−^ solution.

To confirm the antibacterial effect of each compound, the silver ion concentration was measured at different chloride ion concentrations (0, 25, 50, 100, 250, 580, and 1000 ppm) using AAS (Figure 5). At 30–50 Cl^−^/mM, the Ag^+^ concentration of His–Ag was higher than that of Met–Ag or Ag. AAS measurements revealed that the Ag^+^ concentration of Ag was low because of silver chloride (AgCl) formation due to the strong bond between Ag^+^ and Cl^−^. Compared to Ag, Ag–His in particular revealed a higher Ag^+^ ion concentration.

These AAS measurements explained the results of the bacteria growth curves. The concentration of chloride ions in the BHI tested in this study was 25 mM. The AAS data recorded Ag^+^ concentrations at 25 mM were His–Ag > Met–Ag > Ag. Different Ag^+^ concentration likely depended on the higher solubility of silver or silver-amino acid complexes in a solution containing Cl^−^. This higher Ag^+^ concentration gave His–Ag–Mont a longer antibacterial effect.

In our previous study, we tested the antibacterial effect of 10 amino acid–Ag complexes (histidine, methionine, arginine, glycine, alanine, phenylalanine, asparagine, aspartic acid, glutamic acid, and cysteine) and an imidazole–Ag complex in a sea-water based medium containing Cl^−^ [19]. Only histidine, methionine and imidazole formed stable complex with Ag in sea water based medium, and they inhibited bacteria growth. The Ag^+^ concentration of amino acid complex in the medium after bacterial growth was 0.019 mM for Met–Ag complex, 0.016 mM for the His–Ag complex, and less than 0.002 mM for other amino acid complexes (unpublished data). Among the essential amino acids, histidine and methionine have a strong binding energy to Ag^+^ [37]. The Ag–N bond for histidine and Ag–S bond for methionine was also confirmed by our NMR analysis. These strong bonds between Met–Ag and His–Ag may inhibit silver chloride formation due to the weaker bond between Ag^+^ and Cl^−^.

Recently, consumption of silver and silver compounds has increased as antibacterial materials; however, silver-resistant bacteria in Gram-negative pathogens is reported [38,39]. Several periplasmic silver binding proteins (SilE) have been investigated as silver resistance genes. SilE exist at the bacterial cell surface and reduce silver toxicity when a silver ion binds to SilE [11]. Histidine and methionine residues in SilE coordinate to Ag^+^ [39,40]. The ^1^H NMR spectroscopy demonstrated the binding of Ag to the N atom of histidine [11]. Later, the ^1^H NMR spectrum confirmed that Ag^+^ binds to the S atom of methionine [40]. The binding strength changes according to the environment. The histidine-silver ion bond in SilE is stronger in an acidic environment than in a neutral environment [41].

It is known that the distribution of metal complex ion species in solution changes depending on conditions such as coordination concentration, metal ion concentration, temperature, and pH. His–Ag and Met–Ag may have a stable complex formation in Cl^−^ solution due to a relatively strong bond with silver. This may be the reason why lower concentrations of His–Ag and Met–Ag had a longer antibacterial effect. This can be useful for reducing the amounts of silver compounds in dental materials. In this study, His–Ag–Mont had a longer antibacterial effect in BHI medium. AAS measurement revealed that the Ag^+^ of His-Ag complex were higher existence in the Cl^−^ concentration of BHI. The Ag^+^ stability of His–Ag and Met–Ag change depend the concentration of Cl^−^. The Ag^+^ of the Met–Ag complex may be more stable than that of His–Ag complex in a solution of high Cl^−^ concentration. Further study is needed to investigate the antibacterial effect of His–Ag and Met–Ag in different pHs and concentrations of chloride ion for a simulated intra-oral environment.

## 4. Conclusions

This study found that two silver–amino acid-loaded montmorillonites (His–Ag–Mont and Met–Ag–Mont) in dental acrylic resin had a longer antibacterial effect than silver-loaded zeolite and silver-zirconium phosphate. In particular, His–Ag–Mont had a stronger antibacterial effect, which proved the existence of the highest silver ion concentration in a solution containing Cl^−^. Thus, the structure of amino acid–silver strongly affected the antibacterial effect of silver. This study was an exploratory research to find an effective antibacterial agent for dental materials in the existence of chlorine ion in saliva, food and beverages. This study has limitations, however, His–Ag–Mont incorporated with dental acrylic resin may be a candidate as an oral antibacterial agent. Moreover, we also need to confirm the safety in the future.

## Data Availability

The data presented in this study are available on request from the corresponding author.
